# Association of Anemia and Diabetic Retinopathy Among Patients With Type 2 Diabetes Mellitus: Retrospective Cross-Sectional Study

**DOI:** 10.7759/cureus.67995

**Published:** 2024-08-28

**Authors:** Shaikha M AlFalasi, Khuloud A Abdouli, Noura A Aldashti

**Affiliations:** 1 Family Medicine, Dubai Health, Dubai, ARE

**Keywords:** macular edema, type 2 diabetes mellitus, hemoglobin, diabetic retinopathy, anemia

## Abstract

Introduction

Type 2 diabetes mellitus (T2DM) is a common metabolic disorder characterized by the combination of defective insulin secretion and the inability of insulin-sensitive tissues to respond appropriately to insulin. Diabetic retinopathy (DR) is a common microvascular complication that can result in a preventable cause of blindness. Research to determine the prevalence of anemia among diabetic patients is necessary to assess whether treatment practices should be changed. Anemia is a common complication in patients with T2DM and has been associated with the progression of DR. In this study, our aim is to determine the prevalence of DR and its association with hemoglobin levels in patients diagnosed with T2DM in Dubai, UAE.

Methods

In this retrospective cross-sectional study, we extracted the data using electronic medical records. The study was performed over a span of three years in Dubai from 2019 to 2022. A total of 368 T2DM patients were included based on retinal exam findings classified into mild, moderate, severe non-proliferative retinopathy, and proliferative retinopathy. Descriptive statistics were used for categorical (frequency, percentage) and continuous variables (mean, SD), with chi-square/Fisher exact tests for categorical associations, ANOVA for continuous variables, and multiple logistic regression to identify DR risk factors, using OR, 95% CI, and p < 0.05 for significance.

Results

The prevalence of anemia (defined as hemoglobin levels ≤13 mg/dL for men and ≤12 mg/dL for women) was observed in 39.4% of individuals with DR aged between 40 and 88 years; 60.6% of the patients had normal hemoglobin, while 91 individuals (24.7%) exhibited mild anemia, 53 individuals (14.4%) showed moderate anemia, and only one individual (0.3%) had severe anemia. DR grading was as follows: mild non-proliferative DR (16.8%), moderate non-proliferative DR (30.2%), severe non-proliferative DR (13.3%), and proliferative DR (39.7%). Macular edema was present in 59.2% of patients, showing a statistically significant association with more severe DR stages (p < 0.0001). No significant association was found between hemoglobin levels and DR severity (p = 0.568). However, among males, a significant difference in mean hemoglobin levels across DR grades was observed (p = 0.009), with higher hemoglobin levels associated with lower odds of severe DR (OR = 0.775, p = 0.036). Macular edema strongly predicted DR severity, with significant odds ratios across all stages (p < 0.0001).

Conclusions

There is a significant prevalence of anemia among the examined population. DR severity was notably associated with lower hemoglobin levels in males, and macular edema was significantly linked to more severe stages of DR. Vigilant monitoring and integrated care for both anemia and DR are crucial to optimize patient outcomes and mitigate complications. Regular retinopathy screening is essential for early detection and timely intervention, particularly considering the challenges posed by anemia, such as delayed wound healing and increased infection risk post-screening.

## Introduction

Diabetes mellitus, particularly type 2 diabetes mellitus (T2DM), is a common metabolic disorder characterized by mainly the combination of defective insulin secretion by pancreatic β-cells and the inability of insulin-sensitive tissues to respond appropriately to insulin [1]. In 2019, the global diabetes prevalence was 9.3% (463 million people), rising to 10.2% (578 million) by 2030 and 10.9% (700 million) by 2045 [2]. The prevalence is higher in urban (10.8%) than rural (7.2%) places, as well as in high-income (10.4%) than in low-income countries (4.0%) [2]. Gulf Cooperation Council (GCC) nations had the highest prevalence (25.45%), while non-GCC nations had the lowest prevalence (12.69%). The Arab nations with the highest prevalence rates of T2DM include the Kingdom of Saudi Arabia (31.6%), Oman (29.0%), and the United Arab Emirates, Kuwait, and Bahrain, reporting an incidence of 25% each [3]. As the prevalence of T2DM continues to rise globally, it has emerged as a significant public health concern due to its association with various complications such as cardiovascular disease, neuropathy, nephropathy, and retinopathy [4]. Complications associated with diabetes have a detrimental impact on quality of life and mortality and increase healthcare costs [5].

Among the diabetes-associated complications, diabetic retinopathy (DR) stands out as one of the frequent microvascular complications, which can cause acquired yet preventable blindness [6]. It involves damage to the microvasculature of the retina from prolonged exposure to the metabolic changes associated with diabetes [7]. Several factors, including glycemic control, duration of diabetes, age, and albuminuria, can influence the severity and development of DR [5]. Worldwide, the prevalence of DR among diabetic individuals is estimated to be 27.0%, which results in 0.4 million blindness globally [8]. The prevalence of DR in the United Arab Emirates was 19% and significantly affected elderly males [9]. DR progresses through various stages, from mild non‐proliferative to the more serious, sight‐threatening, proliferative stage, characterized by the formation of new blood vessels on the retina [10]. This progression is influenced by several risk factors, including the duration of diabetes, hyperglycemia, hypertension, and dyslipidemia [11]. However, gaining evidence suggests that anemia, characterized by low hemoglobin levels, specifically <13 mg/dL in men and 12 mg/dL in females, may play a crucial part in the onset and progression of DR. Anemia is frequently found in individuals with T2DM, as we found nearly one-quarter of diabetic patients to be anemic [12]. However, it is less often identified related comorbidity that could have an unfavorable effect on the progression of diabetes-related microvascular complications [13]. Patients with hemoglobin levels <12 gm% have a double risk of retinopathy compared to subjects with a normal hemoglobin value. They also have a 5% additional risk of developing proliferative retinopathy [14]. Early recognition and treatment of anemia in the diabetic population decreases morbidity and mortality, which leads to a better quality of life in diabetic subjects [15]. Research to determine the prevalence of anemia among diabetic patients is necessary to assess whether treatment practices should be changed.

In this study, we aim to evaluate the prevalence of DR in anemic patients in T2DM patients as well as the association between the hemoglobin level and DR among T2DM patients in Dubai. Ultimately, it will contribute to the growing body of literature on the intersection of anemia and diabetic complications, emphasizing the significance of comprehensive patient care in diabetes treatment. Thus far, no published studies in the United Arab Emirates, to the best of our knowledge, have been found in this study framework, and it is essential to research the prevalence of anemia among T2DM patients and its association with DR severity staging and to implement health interventions, including early treatment of anemia among these patients.

## Materials and methods

Study design and settings

This retrospective cross-sectional study was conducted using the data extracted from electronic medical records. This database encompasses all outpatient medical claims, including office visits, procedures, and prescribed medications, along with demographic data and laboratory results for patients enrolled in a wide-ranging managed care network in Dubai's healthcare government sector. The subset of data available for this study included 500 patients, and after applying the inclusion and exclusion, the final sample size was 368 within the database. The study was conducted over a span of three years, from January 1, 2019, to January 1, 2022, following a systematic sampling technique.

Study population and inclusion and exclusion

A sample size calculation was performed, assuming a 50% prevalence of the outcome of interest, which provides the most conservative estimate. Using a 95% confidence level and a 5% margin of error, the required sample size was calculated to be approximately 500 participants. This sample size ensures that the study has adequate power to detect statistically significant associations between variables, even in the absence of precise prevalence data. However, after applying the inclusion and exclusion criteria, the final sample was set to 368. We included patients of T2DM with DR of both genders, representing various nationalities, and aged 40 years or older. We excluded pregnant women, patients with type 1 diabetes mellitus, those experiencing acute drops in hemoglobin levels due to bleeding, patients with hemoglobinopathies, and patients with malignancies.

Data collection

Data were obtained from electronic medical records, aggregating patient records from multiple healthcare facilities across Dubai. The dataset included demographic details such as gender and nationality. Clinical parameters, including body mass index (BMI), smoking status, hemoglobin level, DR stages, and glycated hemoglobin (HbA1c) levels, were collected. Medication regimens were classified as mono, dual, or polytherapy (metformin, sulfonylureas or dipeptidyl peptidase-4 (DPP-4) inhibitors, and insulin or glucagon-like peptide 1 (GLP-1) receptor agonists).

Definitions and relevant variable management

The definition of anemia relied on hemoglobin levels ≤13 mg/dL for men and ≤12 mg/dL for women, as per the WHO criteria [16]. To explore the dose-response association between hemoglobin levels and DR, we categorized hemoglobin into four levels in males (<8 mg/dL, 8 to approximately 10.9 mg/dL, 11 to approximately 12.9 mg/dL, and ≥13 mg/dL) and in females (<8 mg/dL, 8 to approximately 10.9 mg/dL, 11 to approximately 11.9 mg/dL, and ≥12 mg/dL), respectively [17].

For analysis purposes, we subdivided patients with DR into mild, moderate, and severe non-proliferative DR (NPDR) and proliferative DR [18]. An experienced ophthalmologist made the diagnosis of DR after a dilated fundus examination. We defined NPDR as the presence of micro-aneurysms, intra-retinal hemorrhages, hard exudates, cotton-wool spots, or macular edema without evidence of retinal or iris neovascularization. We defined proliferative DR as the presence of neovascularization on the optic disc, retina, or iris, with or without vitreous hemorrhage or prior pan-retinal photocoagulation [19,20].

Statistical analysis

The collected data underwent manual data cleaning procedures, and corrections of any errors were made. Data were coded and exported to statistical analyses performed using SPSS version 26.0 (IBM Corp., Armonk, New York) for analysis. The dependent variables were DR, and the independent variables were age, sex, nationality, smoking status, BMI, HbA1C, hemoglobin level, and diabetes mellitus medication.

Descriptive statistics were presented using frequency and percentage for categorical variables and mean and SD for continuous variables. Associations between categorical variables were analyzed using the chi-square test or Fisher exact test, and analyses of continuous variables with more than three groups were done using ANOVA and post hoc tests using Bonferroni. A multiple logistic regression analysis was done to identify the risk factors for DR in the patients. Then, the association between the independent variables and the outcome variable was assessed using the OR and 95% CI for the OR and p < 0.05 as the cut-off point for statistical significance.

Ethical consideration

Ethical approval for this research was received from the Research Ethics Committee of Dubai Health where the research was conducted, with approval number DSREC/RPR/2021/29.

## Results

The study included 368 patients with T2DM and DR, with an average age of 63.12 ± 9.84 years. The gender distribution was nearly equal, with 50.8% males and 49.2% females. The majority of participants were Emirati nationals (83.2%). In terms of BMI, 37.8% were overweight, 37% were obese, and 12.8% had a normal BMI, while a small fraction were underweight or morbidly obese. Smoking status revealed that 31.5% were smokers, 59.8% were non-smokers, and 8.7% had unknown smoking status. Regarding diabetes medications, most participants (70.4%) were on polytherapy, followed by dual therapy (19%) and monotherapy (7.9%). Hemoglobin levels were categorized by gender, with 64.7% of males having normal levels and 8.6% having moderate anemia. The mean hemoglobin level for males was 13.45 ± 1.803 mg/dL. Among females, 56.4% had normal hemoglobin levels, with 20.4% showing moderate anemia. The mean hemoglobin level for females was 12.17 ± 1.511 mg/dL. DR grading was as follows: mild NPDR (16.8%), moderate NPDR (30.2%), severe NPDR (13.3%), and proliferative (39.7%). Additionally, macular edema was present in 59.2% of the patients. HbA1c levels indicated that 42.1% had values less than 7.5%, 44% had values between 7.5 and 10, and 13.9% had values above 10 (Table 1 and Table 2).

**Table 1:** Baseline mean and standard deviation of the age and hemoglobin levels

Risk factor	Mean	± SD
Age	63.12	± 9.847
Hemoglobin level, mg/dL (males)	13.45	± 1.803
Hemoglobin level, mg/dL (females)	12.17	± 1.511

**Table 2:** Baseline characteristics of the study participants BMI: body mass index; DM: diabetes mellitus; HbA1c: glycated hemoglobin; N: number

Risk factors	Category	n (%)
Gender	Male	187 (50.8)
	Female	181 (49.2)
BMI	<18.5 (underweight)	2 (0.5)
	18.5-24.9 (normal)	47 (12.8)
	25-29.9 (overweight)	139 (37.8)
	30-39.9 (obese)	136 (37)
	40 and above (morbidly obese)	26 (7.1)
	Unknown	18 (4.9)
Nationality	Local	306 (83.2)
	Non-local	62 (16.8)
Smoking status	Smoker	116 (31.5)
	Non-smoker	220 (59.8)
	Unknown	32 (8.7)
DM medications	Mono	29 (7.9)
	Dual	70 (19)
	Poly	259 (70.4)
	Unknown	10 (2.7)
Hemoglobin level, mg/dL (males)	Normal	121 (64.7)
	Mild anemia	49 (26.2)
	Moderate anemia	16 (8.6)
	Severe anemia	1 (0.5)
Hemoglobin level, mg/dL (females)	Normal	102 (56.4)
	Mild anemia	42 (23.2)
	Moderate anemia	37 (20.4)
	Severe anemia	0 (0)
Diabetic retinopathy grading	Mild	62 (16.8)
	Moderate	111 (30.2)
	Severe	49 (13.3)
	Proliferative	146 (39.7)
Macular edema	Present	218 (59.2)
	Absent	150 (40.8)
HbA1c	Less than 7.5	155 (42.1)
	7.5-10	162 (44)
	10 and more	51 (13.9)

No significant association was observed between different variables and DR grading except for macular edema, which was observed to be statistically significantly associated with more severe DR stages, with 23.1% having proliferative DR compared to 16.6% without macular edema (p < 0.0001) (Table 3).

**Table 3:** Association between different variables and diabetic retinopathy grading BMI: body mass index; DM: diabetes mellitus; HbA1c: glycated hemoglobin; N: number; SD: standard deviation; *: statistically significant

Variable	Diabetic retinopathy, n (%)				
	Mild	Moderate	Severe	Proliferative	p-value
Sex					
Male	35 (9.5)	50 (13.6)	27 (7.3)	75 (20.4)	0.450
Female	27 (7.3)	61 (16.6)	22 (6)	71 (19.3)	
Smoking status					
Smokers	25 (6.8)	33 (9)	10 (2.7)	48 (13)	0.368
Non-smokers	31 (8.4)	68 (18.5)	33 (9)	88 (23.9)	
Unknown	6 (1.6)	10 (2.7)	6 (1.6)	10 (2.7)	
BMI					
Less than 18.5	0 (0)	0 (0)	0 (0)	2 (0.5)	0.680
18.5-24.9	9 (2.4)	17 (4.6)	6 (1.6)	15 (4.1)	
25-29.9	21 (5.7)	47 (12.8)	19 (5.2)	52 (14.1	
30-39.9	26 (7.1)	39 (10.6)	17 (4.6)	54 (14.7)	
40 and above	4 (1.1)	4 (1.1)	5 (1.4)	13 (3.5)	
DM medications					
Mono	5 (1.4)	9 (2.4)	3 (0.8)	12 (3.3)	0.598
Dual	10 (2.7)	15 (4.1)	11 (3)	34 (9.2)	
Poly	47 (12.8)	83 (22.6)	33 (9)	96 (26.1)	
Unknown	0 (0)	4 (1.1)	2 (0.5)	4 (1.1)	
HbA1c					
Less than 7.5	27 (7.3)	52 (14.1)	17 (4.6)	59 (16)	0.105
7.5-10	32 (8.7)	44 (12)	20 (5.4)	66 (17.9)	
10 and more	3 (0.8)	15 (4.1)	12 (3.3)	21 (5.7)	
Macular edema					
Yes	22 (6)	75 (20.4)	36 (9.8)	85 (23.1)	<0.0001*
No	40 (10.9)	36 (9.8)	13 (3.5)	61 (16.6)	
Age, mean ± SD	64.71 ± 9.12	63.94 ± 10.047	61.02 ± 11.084	62.53 ± 9.472	0.163

**Figure 1:**
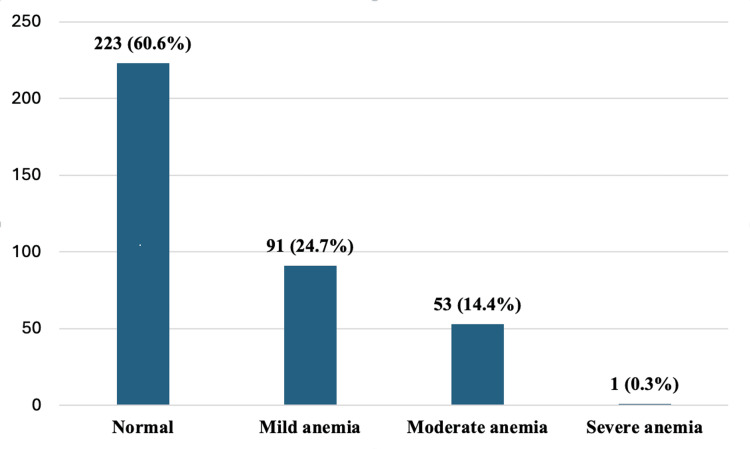
Prevalence of anemia in diabetic retinopathy patients

The overall prevalence of anemia (mild to severe) was 39.40%. The distribution of hemoglobin levels reveals that 223 individuals (60.6%) had normal hemoglobin levels, while 91 individuals (24.7%) exhibited mild anemia, 53 individuals (14.4%) showed moderate anemia, and only one individual (0.3%) had severe anemia (Figure 1).

Table 4 illustrates the distribution of DR grades across varying levels of hemoglobin. Among those with normal hemoglobin levels, the prevalence of mild, moderate, severe, and proliferative DR appears to be distributed across 18.4%, 31.8%, 14.3%, and 35.4%, respectively. Similarly, individuals with mild and moderate anemia showed varying distributions across DR grades, with proliferative retinopathy notably higher in these groups. The p-value of 0.568 suggested that there was no statistically significant association between hemoglobin levels and DR severity.

**Table 4:** Distribution of diabetic retinopathy grades across hemoglobin levels in patients

Hemoglobin level, mg/dL	Diabetic retinopathy grading in overall patients				p-value
	Mild	Moderate	Severe	Proliferative	
Normal	41 (18.4)	71 (31.8)	32 (14.3)	79 (35.4)	0.568
Mild anemia	13 (14.3)	23 (25.3)	13 (14.3)	42 (46.2)	
Moderate anemia	8 (15.1)	17 (32.1)	4 (7.5)	24 (45.3)	
Severe anemia	0 (0)	0 (0)	0 (0)	1 (100)	

The overall prevalence of anemia (defined as hemoglobin levels ≤13 mg/dL for men and ≤12 mg/dL for women) was 30.5% in males and 43.6% in females. The analysis of DR stages in relation to hemoglobin levels showed varied distributions among males and females. The associations were not statistically significant for either gender, with p-values of 0.289 for males and 0.999 for females (Table 5).

**Table 5:** Distribution of diabetic retinopathy severity by hemoglobin levels in male and female patients with type 2 diabetes mellitus

Hemoglobin level (mg/dL)	Diabetic retinopathy in males, n (%)				
	Mild	Moderate	Severe	Proliferative	p-value
Normal (>13 mg/dL)	26 (21.5)	36 (29.8)	19 (15.7)	40 (33.1)	0.279
Mild anemia (11-12.9 mg/dL)	6 (12.2)	10 (20.4)	8 (16.3)	25 (51.0)	
Moderate anemia (8-10.9 mg/dL)	3 (18.8)	4 (25.0)	0 (0)	9 (56.3)	
Severe anemia (<8 mg/dL)	0 (0)	(0)	0 (0)	1 (100)	
Hemoglobin level (mg/dL)	Diabetic retinopathy in females, n (%)				
	Mild	Moderate	Severe	Proliferative	p-value
Normal (>12 mg/dL)	15 (14.7)	35 (34.3)	13 (12.7)	39 (38.2)	0.999
Mild anemia (11-11.9 mg/dL)	7 (16.7)	13 (31.0)	5 (11.9)	17 (40.5)	
Moderate anemia (8-10.9 mg/dL)	5 (13.5)	13 (35.1)	4 (10.8)	15 (40.5)	
Severe anemia (<8 mg/dL)	0 (0)	(0)	0 (0)	0 (0)	

The analysis of hemoglobin levels in relation to DR grades revealed significant findings for males but not for females. Among males, the mean hemoglobin levels were 13.7 ± 1.6 mg/dL for mild DR, 13.7 ± 1.7 mg/dL for moderate DR, 14.1 ± 1.6 mg/dL for severe DR, and 12.9 ± 1.9 mg/dL for proliferative DR, with a statistically significant p-value of 0.009. This indicates a notable difference in hemoglobin levels across DR grades in males. In contrast, for females, the mean hemoglobin levels were 12.1 ± 1.3 mg/dL for mild DR, 12.2 ± 1.7 mg/dL for moderate DR, 12.4 ± 1.4 mg/dL for severe DR, and 12.1 ± 1.5 mg/dL for proliferative DR, with a p-value of 0.896, indicating no significant difference across DR grades in females (Table 6).

**Table 6:** Association between hemoglobin levels and diabetic retinopathy grading SD: standard deviation; *: statistically significant; &: post hoc analysis, showing statistically significant difference

Diabetic retinopathy grade	Male hemoglobin level (mg/dL) mean ± SD	p-value	Female hemoglobin level (mg/dL) mean ± SD	p-value
Mild	13.7 ± 1.6	0.009*	12.1 ± 1.3	0.896
Moderate	13.7 ± 1.7		12.2 ± 1.7	
Severe	14.1 ± 1.6^&^		12.4 ± 1.4	
Proliferative	12.9 ± 1.9^&^		12.1 ± 1.5	

The table presents OR with 95% CI, assessing the association between various factors and DR severity. Notably, higher hemoglobin levels in males are significantly associated with lower odds of severe DR (OR 0.775, 95% CI: 0.611-0.983, p = 0.036). Macular edema, however, shows a strong positive association across all stages (OR range: 2.534-5.035, p < 0.0001), indicating its significant impact on DR severity (Table 7).

**Table 7:** Multinomial logistic regression of diabetic retinopathy grading prediction by different variables (mild as a reference) CI: confidence interval; BMI: body mass index; DM: diabetes mellitus; HbA1c: glycated hemoglobin; OR: odds ratio; SD: standard deviation; *: statistically significant

Variable	Diabetic retinopathy, OR (95%CI)					
	Moderate	p-value	Severe	p-value	Proliferative	p-value
Age	0.992 (0.961, 1.024)	0.618	0.962 (0.925, 1)	0.051	0.977 (0.948, 1.008)	0.144
Sex	0.632 (0.338, 1.182)	0.151	0.947 (0.445, 2.013)	0.887	0.815 (0.448, 1.482)	0.502
Smoking status	0.964 (0.828, 1.121)	0.631	0.996 (0.834, 1189)	0.962	0.930 (0.802, 1.080)	0.343
BMI	0.964 (0.828, 1.121)	0.631	0.996 (0.834, 1.189)	0.962	0.93 (0.802, 1.08)	0.343
DM medications	0.921 (0.709, 1.197)	0.538	1.044 (0.775, 1.407)	0.776	1.121 (0.887, 1.419)	0.339
HbA1c	1.216 (0.86, 1.72)	0.268	1.18 (0.784, 1.775)	0.428	1.047 (0.743, 1.474)	0.793
Categories of hemoglobin level in males	1.006 (0.658, 1.537)	0.98	0.733 (0.382, 1.404)	0.349	1.286 (0.892, 1.855)	0.178
Hemoglobin level in males	0.999 (0.774, 1.29)	0.995	1.139 (0.842, 1.542)	0.399	0.775 (0.611, 0.983)	0.036*
Categories of hemoglobin level in females	1.028 (0.765, 1.382)	0.855	0.98 (0.673, 1.426)	0.916	1.036 (0.776, 1.384)	0.81
Hemoglobin level in females	1.018 (0.754, 1.373)	0.909	1.124 (0.766, 1.651)	0.55	0.987 (0.737, 1.322)	0.932
Macular edema	3.788 (1.968, 7.29)	<0.0001*	5.035 (2.217, 11.435)	<0.0001*	2.534 (1.369, 4.688)	<0.0001*

## Discussion

This study investigated 368 patients with T2DM and DR to investigate the prevalence of anemia and its association with the severity of DR. The findings provided beneficial knowledge about demographic parameters, hemoglobin levels, DR grading, and the influence of macular edema. The study population had an average age of 63.12 years, with most patients having HbA1c values of ≥7.1. The prevalence of DR in older diabetics is higher, and unregulated glycemia and diabetes duration are established risk factors for DR [21]. In our study, the gender distribution was almost equal, with 50.8% males and 49.2% females, providing a balanced representation for the analysis. Most participants were Emirati nationals (83.2%), offering culturally and geographically specific insights into the population. The BMI distribution highlighted a concerning prevalence of overweight (37.8%) and obese (37%) individuals. Scientific studies have highlighted obesity as a significant risk factor for microvascular complications in T2DM [22-24]. In our study, the majority of patients had HbA1c levels greater than 7.5. Higher HbA1c levels, indicative of poorer blood sugar control, were significantly associated with more severe grades of DR [25]. As per Jamshed et al. (2021), there is a direct relationship between DR and HbA1c levels [26]. However, in our study, smoking status, BMI, diabetes medications, and HbA1c levels revealed trends toward an increased prevalence of severe DR, but none reached statistical significance. The lack of significance may be due to the study's sample size and unaccounted confounding variables such as the duration of diabetes. These factors contribute to the non-significant results observed in our study.

The significant association between lower hemoglobin levels and more severe DR in males, as observed in our study, aligns with previous findings that suggest anemia exacerbates the progression of diabetic complications, including DR. For instance, Lee et al. [5] demonstrated that lower hemoglobin levels are linked to an increased risk of DR severity in diabetic patients, emphasizing the role of anemia as a potential modifiable risk factor. Clinically, this suggests that regular monitoring of hemoglobin levels and early intervention to correct anemia may be crucial in managing DR progression, particularly in male patients. Integrating anemia management into diabetes care protocols could help mitigate the risks of vision-threatening complications, thereby improving overall patient outcomes [5].

In the study group, DR grading showed that 16.8% of patients had mild DR, 30.2% had moderate DR, 13.3% had severe DR, and 39.7% had proliferative DR. This distribution of DR severity differs from findings in other GCC countries. For instance, in a study conducted in Saudi Arabia by Bajaber et al. (2021) [27], the prevalence of DR was as follows: 52.6% had mild NPDR, 15.3% had severe NPDR, and only 4.4% had proliferative DR. Our study found a notably higher proportion of proliferative DR (39.7%) compared to Bajaber et al.'s 4.4%, indicating that our population may experience more advanced stages of DR. Additionally, our findings showed a lower prevalence of mild DR (16.8%) compared to the 52.6% reported in Saudi Arabia. An earlier study conducted in Al Ain, UAE, reported 3.8% proliferative DR, which is lower than our proliferative DR prevalence [9].

The overall prevalence of anemia in the present study (defined as hemoglobin levels ≤13 mg/dL for men and ≤12 mg/dL for women) was 39.4%, with 35.3% of males and 43.6% of females meeting the criteria for anemia. This overall prevalence is higher than that of the overall pooled prevalence of anemia among T2DM adult patients, which was 27.0% reported in a recent meta-analysis [28] and in another study conducted in the Emirati population (36.3%), which is close to our prevalence [29]. Also, the prevalence of anemia in our study is higher when compared with the findings of the study conducted in Oman (29.3%) [30] and Yemen (23.8%) [31]. However, our study prevalence was lower than that of Saudi Arabia (42.4%) and 63% in Egypt [32]. Studies of anemia in patients with diabetes were done in various places with different prevalence. Variability in the prevalence can be explained by differences in ethnicity, age of the study participants, and duration of DM.

The analysis of hemoglobin levels in relation to DR severity revealed varied distributions among males and females. Overall, there was no statistically significant association between hemoglobin levels and DR severity (p = 0.568). According to the study by Li et al. (2032), which had a similar age group to our study, there was a negative association between hemoglobin levels and DR after adjusting for several covariates [33]. Anemia is a common complication of diabetes, and in patients with T2DM, it was associated with an increased risk of NPDR (OR = 1.75, 95% CI = 1.18-2.58) and proliferative DR (OR = 3.71, 95% CI = 2.23-6.18) [34], and hemoglobin was the only hematological variable that showed a significant inverse association with the severity of DR (beta-coefficient = -0.52, p = 0.003) [35]. In a Korean study conducted by Lee et al. (2018) [5], they observed varying hemoglobin levels and the prevalence of anemia across different stages of DR, including no DR, mild to moderate NPDR, severe NPDR, and proliferative DR. Their findings indicated a progressive decrease in hemoglobin levels as DR severity advanced, with levels of 14.1 ± 0.1, 13.8 ± 0.1, 13.3 ± 0.2, and 13.5 ± 0.2 g/dL from no DR to PDR, respectively, showing a significant decreasing trend (p for trend < 0.0001). Comparatively, our study also explored the association between hemoglobin levels and DR severity, particularly noting lower hemoglobin levels associated with more advanced DR stages, such as proliferative DR, among males. These findings align with Lee et al.'s observations, suggesting a consistent trend of declining hemoglobin levels with increasing DR severity, albeit in different demographic contexts and populations. However, a closer examination of gender-specific data showed significant findings for males but not for females. Specifically, proliferative DR showed lower hemoglobin levels compared to the non-proliferative group. Additionally, multinomial logistic regression analysis indicated that higher hemoglobin levels in males were significantly linked to lower odds of severe DR (OR 0.775, 95% CI 0.611-0.983, p = 0.036). This contrasts with findings from the Conway et al. (2009) [36] study, where a positive linear relationship between hemoglobin levels and the 18-year incidence of proliferative DR in men was reported (hazard ratio: 1.33; 95% CI: 1.10-1.60; p = 0.003). For women, the referenced study found a quadratic relationship between hemoglobin levels and proliferative DR (p < 0.001), remaining significant after adjusting for covariates (p = 0.04), unlike our study, where no significant association was observed. These differences suggest that while hemoglobin levels are a crucial predictor of DR progression in men, other factors might influence DR severity in women. This highlights the complexity of the relationship between anemia and DR, potentially influenced by hormonal, genetic, and environmental factors that differ between genders. The etiology and pathogenesis of anemia in DM patients are multifactorial. Decreased erythropoietin production is an important cause of the development of anemia in DM patients [37].

Adding more to this context, the findings of a cross-sectional study by Wang et al. (2020) among 901 patients concluded that diabetic kidney disease severity, sporadic estimated glomerular filtration rate, and urine albumin/creatinine ratio were linked to an elevated risk of DR in T2DM patients, and anemia combined influenced these correlations. Hence, improving hemoglobin levels may lower the risk of DR in T2DM patients [34]. Moreover, Traveset et al. (2016) highlighted that low blood oxygen-transport capacity in patients with DR was linked to more severe DR and retinal ischemia. Low hemoglobin levels may play an important role in the onset and course of DR [35].

Diabetic macular edema could happen during any stage of DR, including NPDR and proliferative DR, but is more frequent as the severity of DR increases. Macular edema was present in 59.2% of patients and was significantly associated with more severe DR stages (p < 0.0001). This underscores the critical role of macular edema as a predictor of DR severity, emphasizing the need for early detection and treatment to prevent vision loss. Risk factors for DME and DR are similar.

In this study, we found a statistically significant association between hemoglobin levels and DR in males. However, when considering the overall population, we did not observe a significant association. The differences in our findings compared to existing literature may be attributed to sample size differences and inherent characteristics unique to each study. These factors likely contribute to the variability seen across studies examining this relationship.

Limitations and future research directions

The cross-sectional design limits the ability to establish causal relationships between the identified risk factors and DR severity. The lack of statistical significance for some associations, such as smoking status, HbA1c, and BMI, may be due to unaccounted confounding variables, such as the duration of diabetes and the presence of other comorbidities. Additionally, the study is geographically and culturally specific to the local population, which may limit the generalizability of the findings to other regions or ethnic groups. The small sample size may not be representative, and prevalence rates could differ in larger study populations. The diverse population in the UAE may yield different results compared to established literature, further complicating comparisons. Furthermore, the limited availability of well-established literature on UAE populations may impact the study’s contextual relevance. Another limitation is that anemia can also be influenced by medications, which could confound the association between anemia and DR severity. Despite these limitations, retrospective studies can still provide valuable preliminary evidence or generate hypotheses for further investigation. Future prospective studies with carefully controlled variables and larger sample sizes could offer more definitive insights into the complex relationship between anemia and DR. Future research on the association of anemia and DR should prioritize prospective longitudinal studies to establish temporal relationships and elucidate whether anemia precedes or follows the development of DR. Mechanistic investigations into biological pathways such as oxidative stress, inflammation, and vascular dysfunction linked to anemia and DR are essential to uncovering underlying mechanisms. Diversifying study populations to include different ethnicities, geographical regions, and socioeconomic backgrounds would enhance generalizability. Furthermore, incorporating regular retinopathy screening into standard diabetes care for individuals with anemia can lead to better outcomes through early identification of retinal complications, prompt intervention, and personalized treatment plans. This proactive approach allows healthcare providers to detect any signs of retinopathy early on, enabling timely management to prevent progression and mitigate potential vision loss. By addressing both diabetes and anemia in tandem during screenings, healthcare teams can optimize therapeutic strategies tailored to the specific needs of each patient, thereby enhancing overall health outcomes and quality of life.

## Conclusions

In conclusion, this study reveals a significant prevalence of DR and anemia among the examined population, with a 39.4% overall prevalence of anemia. In males, lower hemoglobin levels were significantly associated with severe proliferative DR, highlighting the importance of anemia management in reducing the risk of advanced DR stages. Additionally, macular edema was strongly linked to more severe DR stages. Vigilant monitoring and integrated care for both anemia and DR are crucial to optimize patient outcomes and mitigate complications. Regular retinopathy screening is essential for early detection and timely intervention, particularly considering the challenges posed by anemia, such as delayed wound healing and increased infection risk post-screening.
